# Impact of Genetic Ancestry on T-cell Acute Lymphoblastic Leukemia Outcomes

**DOI:** 10.21203/rs.3.rs-4858231/v1

**Published:** 2024-08-16

**Authors:** David Teachey, Haley Newman, Shawn Lee, Petri Pölönen, Rawan Shraim, Yimei Li, Hongyan Liu, Richard Aplenc, Shovik Bandyopadhyay, Changya Chen, Zhiguo Chen, Meenakshi Devidas, Caroline Diorio, Kimberly Dunsmore, Omar Elghawy, Amira Elhachimi, Tori Fuller, Sumit Gupta, Junior Hall, Andrew Hughes, Stephen Hunger, Mignon Loh, Zachary Martinez, Michael McCoy, Cassidy Mullen, Stanley Pounds, Elizabeth Raetz, Theresa Ryan, Anna Seffernick, Gongping Shi, Jonathan Sussman, Kai Tan, Lahari Uppuluri, Tiffaney L Vincent, Ruth Wang'ondu, Lena Winestone, Stuart Winter, Brent Wood, Gang Wu, Jason Xu, Wenjian Yang, Charles Mullighan, Jun Yang, Kira Bona

## Abstract

The influence of genetic ancestry on biology, survival outcomes, and risk stratification in T-cell Acute Lymphoblastic Leukemia (T-ALL) has not been explored. Genetic ancestry was genomically-derived from DNA-based single nucleotide polymorphisms in children and young adults with T-ALL treated on Children’s Oncology Group trial AALL0434. We determined associations of genetic ancestry, leukemia genomics and survival outcomes; co-primary outcomes were genomic subtype, pathway alteration, overall survival (OS), and event-free survival (EFS). Among 1309 patients, T-ALL molecular subtypes varied significantly by genetic ancestry, including increased frequency of genomically defined ETP-like, MLLT10, and BCL11B-activated subtypes in patients of African ancestry. In multivariable Cox models adjusting for high-risk subtype and pathways, patients of Admixed American ancestry had superior 5-year EFS/OS compared with European; EFS/OS for patients of African and European ancestry were similar. The prognostic value of five commonly altered T-ALL genes varied by ancestry – including *NOTCH1* , which was associated with superior OS for patients of European and Admixed American ancestry but non-prognostic among patients of African ancestry. Furthermore, a published five-gene risk classifier accurately risk stratified patients of European ancestry, but misclassified patients of African ancestry. We developed a penalized Cox model which successfully risk stratified patients across ancestries. Overall, 80% of patients had a genomic alteration in at least one gene with differential prognostic impact by genetic ancestry. T-ALL genomics and prognostic associations of genomic alterations vary by genetic ancestry. These data demonstrate the importance of incorporating genetic ancestry into analyses of tumor biology for risk classification algorithms.

## Introduction

Despite improvements in survival for many pediatric cancers, disparities persist such that children of Black race and Hispanic ethnicity continue to have inferior outcomes as compared to non-Hispanic White children.[1,2] Race and ethnicity are social constructs associated with exposure to social determinants of health (SDOH) such as poverty, structural racism,[3] and unmet basic resource needs—in part explaining disparate outcomes.[4] Although there is significant heterogeneity within socially constructed racial and ethnic groups, self-identified race and ethnicity may correlate with an individual's ancestral origins.[5,6] Genetic ancestry can be defined by stretches of the genome inherited from familial predecessors and determined by comparing hundreds of thousands of single nucleotide polymorphisms (SNPs) with frequency of SNPs from global reference populations.[5,7,8] Abundant literature demonstrates associations between genetic ancestry and cancer biology.[5,9,10] In pediatric B-cell acute lymphoblastic leukemia (B-ALL), for example, individuals with Admixed American ancestry who frequently identify as having Hispanic ethnicity are more likely to harbor high risk *CRLF2* rearranged Ph-like subtype due to higher prevalence of the germline *GATA3* risk allele.[11] T-cell ALL (T-ALL) is more biologically heterogeneous in terms of activating drivers and mutational landscape as compared with B-ALL.[12] Whether tumor biology and its prognostic relevance are impacted by genetic ancestry in T-ALL has not been previously explored.

Increasingly, cancer treatment utilizes differences in disease biology to risk stratify patients. Tumor biology may allocate patients to novel or intensified therapy for patients at high risk of relapse, and de-escalate treatment for lower-risk patients.[13] To date, genomic risk stratification in T-ALL has been rudimentary, and whether recently identified risk biomarkers have uniform prognostic value for all patients remains unclear. Identifying the impact of genetic ancestry on biology, survival outcomes, and prognostic utility of previously identified biomarkers is imperative for optimal and equitable risk allocation and treatment.

The largest cohort of T-ALL comprehensively sequenced to date—over 1300 children, adolescents, and young adults (CAYA)—recently identified 15 unique T-ALL subtypes with nearly 60% of clonal leukemic drivers occurring due to alterations in non-coding regions.[14] Using this multiomic AALL0434 dataset, we examined the influence of genetic ancestry on T-ALL genomics and survival outcomes and evaluated whether the prognostic utility of specific biomarkers varied by genetic ancestry.

## Results

The analytic cohort included 1309 patients (median 9 years, interquartile range 5-13, range 1-29) with T-ALL treated on the Children's Oncology Group (COG) clinical trial AALL0434. The distribution of categorical genomically-defined ancestry: 58% European, 15% Admixed American, 11% African, 3% East Asian, 2% South Asian, and 12% Other. Clinical features including central nervous system (CNS) status, diagnostic white blood cell count (WBC), and end induction measurable residual disease (MRD) were similar across ancestries (**Table 1**). Patients of African and Admixed American ancestry were more likely to have Medicaid-only insurance as compared with patients of European ancestry (49.7%, 46.9%, 20.3%, respectively). Patients of African ancestry were more likely to harbor ETP and near-ETP immunophenotype T-ALL as compared with patients of European ancestry (11.9% vs. 7.4%).

Association of genetic ancestry with T-ALL subtype and pathway alterations

*Subtype*

Overall, T-ALL genomic subtypes varied across genetic ancestries (P<0.001, **Figure 1A**). The newly identified high-risk ETP-like subtype, as well as MLLT10 and BCL11B-activated subtypes accounted for greater proportion of T-ALL cases among CAYA of African ancestry as compared with European ancestry (ETP-like OR 2.49, 95% CI 1.45-4.28 **Table 2;** 29.3% vs 14.5% **Table S1**). Within ETP-like subtype, there was also variation in subtype classifying drivers by ancestry (**Extended Data Fig 1**). Patients of South Asian and East Asian ancestry had notable differences in predominant subtypes compared with European ancestry, but interpretation was limited given small numbers. Continuous ancestry analyses mirrored associations in primary findings (**Extended Data Table 1**). There were also differences in driver gene alterations, with *TLX3* and *TLX1* being more common drivers in European, and *MLLT10* and *MED12* more common in patients of African ancestry (**Figure 1B**).

*Pathways*

African ancestry was associated with decreased odds of expression of Cell cycle (OR 0.58, 95% CI 0.40-0.87), Notch (OR 0.59, 95% CI 0.41-0.87) and Ribosome pathway alterations (OR 0.28, 95% CI 0.11-0.61), and increased odds of Ras pathway alteration (OR 1.68, 95% CI 1.07-2.58; **Table 2, Table S2**) as compared to European ancestry. Similar results were observed with continuous ancestry analyses (**Extended Data Table 2)**.

Association of genetic ancestry with survival outcomes

Kaplan-Meier estimates of 5-year overall survival (OS) were 94.7% (95% CI 90.3-97.1), 89.8% (95% CI 83.4-93.8), and 88.7% (95% CI 86.2-90.8) for patients of Admixed American, African, and European ancestry, respectively (**Figure 2**). Individuals of Admixed American ancestry had significantly superior OS as compared with European (P=0.02) whereas no significant difference was observed between African and European ancestry (P=0.74). Event free survival (EFS) was superior among patients of Admixed American ancestry as compared with European ancestry but did not reach statistical significance (5-year EFS 88.8% vs 81.4%, P=0.09). Results were confirmed in multivariable Cox models, adjusting for covariates, subtype, and pathway alterations, with Admixed American ancestry having a lower hazard as compared with European ancestry (adjusted HR OS=0.51, 95% CI 0.28-0.95; adjusted HR EFS 0.64, 95% CI 0.41-0.98; **Extended Data Table 3, Extended Data Table 4**). Types of events did not differ between ancestral groups (**Table S3**).

Genomic biomarkers by genetic ancestry

*NOTCH pathway*

NOTCH is the most commonly dysregulated pathway in T-ALL with alterations identified in this cohort in *NOTCH1* (n=903, 78%), *FBXW7* (n=285, 22%), *ZMIZ1* (n=7, 1%). Overall, patients with NOTCH pathway alterations experienced significantly superior OS/EFS as compared to those without; however, when stratified by ancestry NOTCH alteration conferred favorable prognosis for patients of European and Admixed American ancestry but not for patients of African ancestry (**Figure 3,** left panel). Furthermore, we observed differential prognostic value for *NOTCH1* and *FBXW7* by ancestry (**Extended Data Fig 5**): *NOTCH1* conferred favorable prognosis for patients of European and Admixed American ancestry but not for patients of African ancestry; *FBXW7* conferred favorable prognosis for patients of Admixed American ancestry only. In terms of frequency, patients of African ancestry were less likely to harbor alterations in NOTCH pathway overall, with lower frequency of *NOTCH1* mutations and similar frequency of *FBXW7* mutations as compared with patients of European and Admixed American ancestry (**Table S4**).

We recently observed that different types of *NOTCH1* alterations have differential prognostic impact—intragenic deletion and intronic SNV/indel associated with negative outcomes; indel, SNV, and stop/frameshift/splice mutations associated with favorable outcomes.[14] Herein we observed a greater proportion of deleterious *NOTCH1* alterations among patients of African ancestry as compared with European (13% vs 6% P= 0.04)**.** Furthermore,*NOTCH1* alterations that were favorable in the overall cohort overall and among patients of European ancestry did not confer similarly favorable EFS among patients of African ancestry (**Extended Data Fig 2**) – in part explaining the non-prognostic value of *NOTCH1* alterations in this group. Finally, in a comparison of *NOTCH1* and *FBXW7*

coding mutation type (frameshift, missense, nonsense), we observed a greater proportion of frameshift and smaller proportion of missense mutations in *NOTCH1* among patients of African ancestry as compared with European and Admixed American (frameshift 54%, 31%, 33%, respectively) with similar proportions of *FBXW7* coding mutations (**Extended Data Fig 3, Extended Data Fig 4**).

*Group for Research on Adult Lymphoblastic Leukemia (GRAALL) risk classifier*

Studies by GRAALL cooperative group identified a prognostic risk classifier, with mutations in *NOTCH1/FBXW7* in the absence of *NRAS/KRAS* or *PTEN* mutations portending favorable outcomes, and conversely, absence of *NOTCH1/FBXW7* and presence of *NRAS/KRAS/PTEN* alterations distinguishing patients with poor outcomes.[15,16] We applied this gene classifier—*NOTCH1/FBXW7* (N/F), *NRAS/KRAS/PTEN* (R/P)—to our cohort and examined its association with survival stratified by genetic ancestry. Among patients of European and Admixed American ancestry, the GRAALL classifier successfully differentiated survival outcomes; however, patients of African ancestry were misclassified (**Figure 3,** center panel). Examining all genes in this classifier separately, a difference in prognostic value by ancestry was observed for *NOTCH1, PTEN* and *NRAS/KRAS*; for example, *NRAS/KRAS* alterations were significantly deleterious only for individuals of African ancestry (**Extended Data Fig 5**).

Among altered genes/regions in at least 5% of patients per ancestral group, we further explored prognostic value by genetic ancestry. A difference in prognostic association was observed for 5 of the top 14 most commonly altered genes/regions in T-ALL, including: *NOTCH1, PHF6, PTEN, NRAS/KRAS* and loss of chromosome 6q. In contrast, there were no differences for *CDKN2A, FBXW7, DNM2, LEF1, MYB, MYC, WT1, USP7, IL7R* (**Figure 4**). No single genomic alteration was prognostic across all ancestral groups.

*Penalized Cox regression model risk classifier*

Our group recently published a novel penalized Cox regression model incorporating clinical variables (MRD, CNS status, WBC), genetic subtype, and specific genomic alterations to risk stratify patients, with resulting 5-year EFS ranging from 65% (highest risk) to 97% (lowest risk).[14] Unlike the GRAALL-classifier, this model-based classifier successfully risk stratified all patients, with similar EFS ranges for each risk group across ancestries and as compared with the cohort overall (**Figure 3,** right panel; All patients $P < 0.001$, European $P<0.001$, Admixed American $P=0.01$, African $P=0.02$).

### Discussion

We observed significant differences in leukemia biology by genetic ancestry in the largest cohort of patients with T-ALL sequenced to date. The greatest differences in T-ALL subtype and pathway deregulation were observed between patients of African as compared with European ancestry. We also found that the prognostic value of individual genomic alterations—including the Notch pathway—and a previously published five-gene risk classifier[17] varied by genetic ancestry. Specifically, in this cohort the five-gene classifier successfully stratified patients of European ancestry into high and low-risk groups but failed to accurately risk-stratify patients of African ancestry. In contrast to our prior findings in B-ALL,[9,18] we found significantly superior survival among patients of Admixed American ancestry, and similar survival among patients of African compared to European ancestry. Taken together, these findings suggest the immediate need to incorporate analysis of genetic ancestry into risk stratification algorithms on phase three clinical trials.

This is the first study to explore the impact of genetic ancestry in T-ALL incorporating tumor genomics. In pediatric B-ALL, Admixed American ancestry is associated with greater prevalence of CRLF2 rearrangement and African ancestry is associated with greater prevalence of TCF3::PBX1 and less hyperdiploidy.[9] In adult cancers, women of African ancestry are more likely to have triple-negative hormone receptor breast cancer as compared with European ancestry,[19] and individuals of Asian ancestry with non-small cell lung cancer are more likely to harbor pathogenic alterations in EGFR.[20] There have been two publications in acute myeloid leukemia (AML) suggesting differences in prognostic association of genetic alterations by social race, but without analysis of genetically defined ancestry.

21,22

Herein we demonstrate not only differences in the frequency of genetic alterations by genetic ancestry in a large pediatric population, but also that the prognostic value of common genetic alterations—including NOTCH1—differ by genetic ancestry. The implication of this finding is that if *NOTCH1* were utilized to risk stratify patients, it might correctly risk stratify patients of European ancestry but misclassify patients of African ancestry—a finding highly relevant to clinical trial design and patient care. A similar finding has been reported in adults with solid tumors among whom *MGA* alterations were associated with superior OS among patients of European ancestry and inferior OS among patients of Asian ancestry.[23] To our knowledge, this is the first report of differential biomarker prognostication between ancestral groups in a hematologic malignancy.

Survival outcomes in pediatric oncology are influenced by biologic phenomena and SDOH. Most prior literature has focused on racial and ethnic outcome disparities associated with adverse SDOH including structural racism, poverty, and access to quality health care. Race and ethnicity are social constructs without biologic basis, yet with some association to genetic ancestral origins.[6,24,25] In contrast to B-ALL, we observed superior outcomes for CAYA of Admixed American ancestry and similar outcomes for CAYA of African ancestry as compared to those of European ancestry.[9] This was not explained by a predominance of low-risk leukemia genomics. There may be complex germline variants, more prevalent among patients of Admixed American or African ancestry with T-ALL, such that chemotherapy metabolism or drug sensitivity overcome impacts of adverse SDOH for these patients, warranting further investigation. Concurrent evaluation of both SDOH and biologic drivers of outcome disparities is essential inform health care delivery interventions and advance equity.

We acknowledge limitations in our study. Prior literature among CAYA with cancer suggests that patients of socially minoritized race and ethnicity are more likely to be treated off study.[26,27] Thus, our cohort may not represent the full distribution of all genomic alterations, particularly among patients with greater proportions of non-European ancestry. Although we observed differences in biology and survival patterns among patients of East Asian and South Asian ancestries, we were unable to draw conclusions due to limited sample size, warranting further investigation. Additionally, the penalized cox regression model requires validation in global populations.

Most children in the United States with newly diagnosed cancer are treated on cooperative group clinical trials or with treatment regimens that became standard of care based on preceding trial results. Increasingly, frontline trials rely on prognostic biomarkers for risk stratification.[28] Given that patients of minoritized social race and ethnicity who are more likely to have non-European genetic ancestry already experience disparities in cancer outcomes, attention to the clinical implementation of genomic biomarkers in treatment decision-making is essential to promote health equity. Our results suggest that ensuring equivalent utility of genomic risk classifiers across ancestries is essential for appropriate risk stratification. Without this critical step, we risk misclassifying patients into high- or low-risk groups, potentially leading to undertreatment and increased risk of relapse, or overtreatment and unnecessary toxicity. Additionally, the validity of statistical analysis in phase three clinical trials relies on appropriate classification of children into high- and low-risk groups. Misclassification due to differential utility of genomic classifiers by ancestry has the potential to directly impact the interpretation of clinical trial results. These data suggest that risk classifiers should be examined by genetic ancestry to ensure equivalent efficacy before implementation in clinical trials.

## Methods

Study Population

The participants included in this study were enrolled on the Children's Oncology Group (COG) clinical trial AALL0434 (NCT04408005) conducted from 2007 to 2014.[29] CAYA with newly diagnosed T-ALL ages 1 to 31 years old were eligible. All subjects with T-ALL were required to enroll on a companion classification study for biobanking and risk stratification, AALL03B1 (NCT00482352) or AALL08B1 (NCT01142427). These trials were approved by the National Cancer Institute Cancer Therapy Evaluation Program, the Pediatric Central Institutional Review Board (IRB), and participating center IRBs. Written informed consents for trial enrollment, specimen banking, and future research were obtained from caregivers and/or patients at the time of original COG study enrollment. Study design and results of AALL0434 have been published.[29-31]

Exposure: genetic ancestry

DNA-based genetic ancestry was the primary exposure of interest. Individual genetic ancestral composition was based on comparison of every patient's genotypes and allele frequencies in reference populations (1000 genomes project).[8] Genome-wide single nucleotide polymorphisms (SNP) with a minor allele frequency > 1% were randomly selected and the fraction of genome derived from a reference population was estimated using the maximum likelihood method with the sum of coefficients from 5 populations assumed to sum to 100%.[32] For every patient, data from the germline SNP genotyping from the Infinium Omni2.5Exome

BeadChip was used in ancestry estimation. For categorization of patients into categorical ancestral groups, definitions were consistent with previously published methods: individuals were classified by composition of genetic ancestry defined as African (African > 70%), East Asian (East Asian > 90%), Admixed American (Amerindian > 10% and Amerindian > African), South Asian (South Asian > 70%), European (European > 90%), and patients who did not meet these thresholds defined as Other.[5,9,33] Individuals with ancestry from indigenous populations of North American and/or South American often have a more heterogenous composition of ancestry-specific SNPs.[34] Thus, these individuals are referred to as having "Admixed American" ancestry.

Outcome: subtype and pathway alteration

Recent integrated (WGS/WES/RNA seq) genomic analysis identified 15 unique T-ALL subtypes with distinct genomic drivers and oncogene expression (**Table S4**).[14] The ETP-like subtype is driven by alterations in a set of genes encoding regulators of hematopoietic stem cell development and is immunophenotypically variable.[14] Coding and non-coding alterations in T-ALL can also be grouped into 17 distinct aberrant signaling pathways.[14] Subtypes, dysregulated pathways, and driver gene alterations were examined for association with genetic ancestry, and as prognostic biomarkers for survival outcomes.

Outcome: survival

Overall survival (OS) was defined as time from date of enrollment to date of death from any cause or censored at last contact. Event free survival (EFS) was defined as time from enrollment to first event (induction failure, induction death, relapse, second malignant neoplasm, or remission death) or date of last contact.[29]

Covariates

Patient characteristics examined for potential confounding included age, sex, insurance status, central nervous system (CNS) status, diagnostic white blood cell count (WBC), day 29 measurable residual disease (MRD), and trial arm.[29] Early T-cell Precursor (ETP) status by immunophenotype (distinct from ETP-like genomic subtype) was also examined as a potential confounder. ETP was defined by central evaluation of diagnostic samples from 1140 patients utilizing the definition of ETP T-ALL as CD8- and CD1a- (<5% positive), weak CD5 expression, and expression of one or myeloid and/or stem cell markers (>25%). Near ETP was defined with this same immunophenotype but stronger CD5 expression.[35]

Statistical analysis

Baseline characteristics were summarized by descriptive statistics. Chi-square or Fisher's exact test were conducted for the association of categorical ancestry with subtype, driver genes, and pathway alterations. All regression models considered European ancestry as the reference group. Associations between ancestry and subtype were modeled using a multinomial regression with TAL1 DP-like as the reference group, as it was the most common. Association of ancestry and pathway alterations were modeled using separate logistic regressions for individual pathways. The Holmes test corrected for multiple comparisons.

Association of biologic subtype and genetic ancestry as a continuous variable were assessed with a two-step procedure. First, we assessed whether there was an overall ancestry related difference in T-ALL subtype. We performed an overall likelihood ratio test, a chi-square test comparing a multinomial regression model without any ancestry variable to a model including all 4 ancestries as continuous variables (European ancestry left out as the reference group). If there was an overall association, step two then examined the association of each ancestry with subtype. For continuous ancestry analysis, we present odds ratios for every 25% increase in a non-European ancestry with European as the reference group, and with TAL1-DP as the reference subtype given it was the most common among all ancestral groups.[9] Thus, an odds ratio associated with 25% increase in African ancestry refers to the increase or decrease in odds of a given T-ALL subtype expression when African ancestry increases with concurrent decrease in European ancestry and all other ancestries held constant. For assessment of association of continuous genetic ancestry and pathway alterations, a separate logistic regression model was constructed for each individual pathway. The same process as subtype analysis was performed for pathway analysis except a logistic regression model for each individual pathway was constructed. The Holmes test was used to correct for multiple comparisons.

OS/EFS were censored at 5-years; few documented events subsequently occurred. Kaplan-Meier curves were plotted by ancestry and compared using log rank tests. Univariable and multivariable Cox proportional hazard regression models were used to estimate hazard ratios (HR). Covariates associated with exposure (P<0.2 or absolute difference of ≥10%) and outcome (P<0.2 or HR ≥1.5 or ≤0.67) were included in the multivariable model; age and sex were included regardless of statistical association.

In the *post hoc* analysis, we examined prognostic utility of pathway alterations, genetic variants, and two previously reported risk classifiers to evaluate the combined effect of T-ALL biology and genetic ancestry on survival outcomes. Although many studies have proposed genomic classifiers for risk stratification in T-ALL, few classifiers have been applied across several cohorts. An exception is a five-gene risk classifier, originally identified by Trinquand et al from The Group of Research on Adult Acute Lymphoblastic Leukemia (GRAALL-2003 and GRAALL-2005)[15] and subsequently applied to two European pediatric cohorts (FRALLE2000T, UKALL2003).[17,36] This classified individuals based on *NOTCH1, FBXW7*, *NRAS/KRAS*, and *PTEN* alterations. Therefore, we selected this classifier to examine utility by genetic ancestry. We also examined a recently published penalized Cox regression model with clinical and genomic variables.[14] We then applied these risk classifiers and stratified by genetic ancestry to evaluate efficacy across different ancestral groups.

Analyses used Stata Be 17 and R, version 4.0.4 (R Group for Statistical Computing).

### Declarations

Competing interest statement

D.T.T. received research funding from BEAM Therapeutics, NeoImmune Tech and serves on advisory boards for BEAM Therapeutics, Janssen, Servier, Sobi, and Jazz. D.T.T. has multiple patents pending on CAR-T. C.G.M. serves on scientific advisory board and honoraria for Illumina, and received research funding from Pfizer, equity from Amgen and royalties from Cyrus. E.A.R. received research funding from Pfizer and serves on a DSMB for BMS.

Funding

K12CA076931-24 (H.N., CJD), Gabriella Miller Kids First X01HD100702 (D.T.T., C.G.M., P.P., M.L.L., S.P.H., S.W., E.A.R., B.L.W., M.D., S.P.B., K.P.D., J.J.Y.), R03CA256550 (D.T.T., C.G.M., P.P., M.L.L., S.P.H., S.W., E.A.R., B.L.W., M.D., S.P.B., K.P.D., J.J.Y.), Alex's Lemonade Stand Foundation (D.T.T., K.T., S.P.H., CJD), the Leukemia and Lymphoma Society (D.T.T.), Singapore NMRC (SHRL), Singapore NUHS NCSP (SHRL), Hyundai Hope on Wheels (D.T.T., K.T., R.S.), R01CA193776 (D.T.T., B.W., K.T., C.G.M., S.P.H., J.J.Y., R.S., M.D.), U10CA180886 (D.T.T., M.L.L.), R01CA264837 (D.T.T., J.J.Y., C.G.M., K.T., B.W., R.S.), U24CA114766 (D.T.T., M.L.L.), U24CA196173 (D.T.T.), U10CA180899 (D.T.T), St. Baldricks Research Foundation (D.T.T), Pennsylvania Department of Health (D.T.T.), the Harrison Willing Memorial Research Fund (D.T.T), The Invisible Prince Foundation (D.T.T), the Aiden Everett Davies Innovation Fund (D.T.T), American Lebanese and Syrian Associated Charities of St. Jude Children's Research Hospital (C.G.M), The St Jude Chromatin Collaborative (C.G.M), P30CA021765 (C.G.M.), R35CA197695 (C.G.M.), U54CA243124 (C.G.M.), Canadian Institute for Health Research (CJD). The content is solely the responsibility of the authors and does not necessarily represent the official views of the National Institutes of Health.

### Tables

| Table 1. Demographics and Clinical Characteristics by Genetic Ancestry | | | | | | | | |
|---|---|---|---|---|---|---|---|---|
| | Total | African | Admixed American | East Asian | European | South Asian | Other | P value |
| | N=1,309<br>n (%) | N=143<br>n (%) | N=194<br>n (%) | N=42<br>n (%) | N=753<br>n (%) | N=20<br>n (%) | N=157<br>n (%) | |
| Age, years | 37 (2.8%) | 4 (2.8%) | 4 (2.1%) | 1 (2.4%) | 20 (2.7%) | 0 (0.0%) | 8 (5.1%) | 0.17 |
| Less than 2 | 225 (17.2%) | 19 (13.3%) | 31 (16.0%) | 9 (21.4%) | 138 (18.3%) | 4 (20.0%) | 24 (15.3%) | |
| 2 to 4.99 | 446 (34.1%) | 52 (36.4%) | 77 (39.7%) | 5 (11.9%) | 251 (33.3%) | 9 (45.0%) | 52 (33.1%) | |
| 5 to 9.99 | 601 (45.9%) | 68 (47.6%) | 82 (42.3%) | 27 (64.3%) | 344 (45.7%) | 7 (35.0%) | 73 (46.5%) | |
| 10 and above | | | | | | | | |
| Sex | 337 (25.7%) | 41 (28.7%) | 50 (25.8%) | 12 (28.6%) | 184 (24.4%) | 9 (45.0%) | 41 (26.1%) | 0.37 |
| Female | 972 (74.3%) | 102 (71.3%) | 144 (74.2%) | 30 (71.4%) | 569 (75.6%) | 11 (55.0%) | 116 (73.9%) | |
| Male | | | | | | | | |
| Insurance status | 373 (28.5%) | 71 (49.7%) | 91 (46.9%) | 8 (19.0%) | 153 (20.3%) | 3 (15.0%) | 47 (29.9%) | <0.001 |
| Medicaid-only | 936 (71.5%) | 72 (50.3%) | 103 (53.1%) | 34 (81.0%) | 600 (79.7%) | 17 (85.0%) | 110 (70.1%) | |
| Other | | | | | | | | |
| CNS status | 1205(92.1%) | 131 (91.6%) | 179 (92.3%) | 39 (92.9%) | 689 (91.5%) | 19 (95.0%) | 148 (94.3%) | 0.94 |
| CNS 1 or 2 | 100 (7.6%) | 12 (8.4%) | 15 (7.7%) | 3 (7.1%) | 60 (8.0%) | 1 (5.0%) | 9 (5.7%) | |
| CNS 3 | 4 (0.3%) | 0 (0.0%) | 0 (0.0%) | 0 (0.0%) | 4 (0.5%) | 0 (0.0%) | 0 (0.0%) | |
| Missing | | | | | | | | |
| WBC (X1000/uL) | 553 (42.2%) | 61 (42.7%) | 89 (45.9%) | 21 (50.0%) | 299 (39.7%) | 7 (35.0%) | 76 (48.4%) | 0.22 |
| <50 | 756 (57.8%) | 82 (57.3%) | 105 (54.1%) | 21 (50.0%) | 454 (60.3%) | 13 (65.0%) | 81 (51.6%) | |
| >= 50 | | | | | | | | |
| MRD, day 29 | 529 (40.4%) | 62 (43.4%) | 85 (43.8%) | 16 (38.1%) | 282 (37.5%) | 9 (45.0%) | 75 (47.8%) | 0.27 |
| >=0.01 | 773 (59.1%) | 79 (55.2%) | 109 (56.2%) | 26 (61.9%) | 466 (61.9%) | 11 (55.0%) | 82 (52.2%) | |
| <0.01 | 7 (0.5%) | 2 (1.4%) | 0 (0.0%) | 0 (0.0%) | 5 (0.7%) | 0 (0.0%) | 0 (0.0%) | |
| Missing | | | | | | | | |
| T-ALL ETP immunophenotype | 110 (8.4%) | 17 (11.9%) | 9 (4.6%) | 5 (11.9%) | 56 (7.4%) | 3 (15.0%) | 20 (12.7%) | 0.07 |
| ETP | 168 (12.8%) | 25 (17.5%) | 29 (14.9%) | 7 (16.7%) | 82 (10.9%) | 2 (10.0%) | 23 (14.6%) | |
| Near-ETP | 862 (65.9%) | 82 (57.3%) | 134 (69.1%) | 27 (64.3%) | 508 (67.5%) | 12 (60.0%) | 99 (63.1%) | |
| Non-ETP | 169 (12.9%) | 19 (13.3%) | 22 (11.3%) | 3 (7.1%) | 107 (14.2%) | 3 (15.0%) | 15 ( 9.6%) | |
| Unknown | | | | | | | | |
| Trial Arm* | 317 (24.2%) | 35 (24.5%) | 40 (20.6%) | 10 (23.8%) | 184 (24.4%) | 5 (25.0%) | 43 (27.4%) | 0.52 |
| Arm A | 134 (10.2%) | 15 (10.5%) | 28 (14.4%) | 4 (9.5%) | 69 (9.2%) | 1 (5.0%) | 17 (10.8%) | |
| Arm B | 390 (29.8%) | 41 (28.7%) | 56 (28.9%) | 7 (16.7%) | 236 (31.3%) | 7 (35.0%) | 43 (27.4%) | |
| Arm C | 197 (15.0%) | | 32 (16.5%) | 13 (31.0%) | 105 (13.9%) | 1 (5.0%) | | |
| Arm D | 269 (20.6%) | | 38 (19.6%) | | | | | |

| | | | | | | | |
|---|---|---|---|---|---|---|---|
| Standard Induction | 2 (0.2%) | 26 (18.2%) | 0 (0.0%) | 8 (19.0%) | 157 (20.8%) | 6 (30.0%) | 20 (12.7%) |
| Missing | | 26 (18.2%) | | 0 (0.0%) | 2 (0.3%) | 0 (0.0%) | 34 (21.7%) |
| | | 0 (0.0%) | | | | | 0 (0.0%) |

*Arm A = Capizzi methotrexate, Arm B=Capizzi methotrexate + Nelarabine, Arm C=High dose methotrexate, Arm D=High dose methotrexate + nelarabine, Standard induction (not randomized); CNS denotes central nervous system, WBC denotes White Blood Cell, MRD denotes bone marrow minimal residual disease, ETP denotes Early T-cell Precursor

Table 2. Association of Genetic Ancestry with Biologic Subtype (TAL1 DP-like as reference subtype) and Pathway Alterations[a]

| Subtype | | | | | | | | | | |
|---|---|---|---|---|---|---|---|---|---|---|
| | | **European** | **African** | | **Admixed American** | | **East Asian** | | **South Asian** | |
| **Subtype** | Total number of patients | | OR (95% CI) | P value | OR (95% CI) | P value | OR (95% CI) | P value | OR (95% CI) | P value |
| TAL1 DP-like | 296 | Reference | Reference | | Reference | | Reference | | Reference | |
| ETP-like | 235 | | 2.49 (1.45-4.28) | <0.001 | 1.23 (0.75-2.02) | 0.41 | 1.51 (0.64-3.53) | 0.35 | 1.31 (0.35-5.00) | 0.69 |
| TAL1 AB-like | 219 | | 1.29 (0.71-2.36) | 0.40 | 1.30 (0.80-2.09) | 0.29 | 0.88 (0.34-2.31) | 0.80 | NA | NA |
| TLX3 | 212 | | 0.58 (0.29-1.20) | 0.14 | 0.88 (0.53-1.45) | 0.61 | 0.79 (0.24-1.80) | 0.42 | 0.79 (0.19-3.36) | 0.75 |
| NKX2-1 | 77 | | 0.63 (0.23-1.70) | 0.36 | 0.56 (0.25-1.26) | 0.16 | NA | NA | 2.70 (0.70-10.4) | 0.15 |
| TLX1 | 71 | | 0.35 (0.10-1.19) | 0.09 | 0.33 (0.12-0.86) | 0.02 | NA | NA | NA | NA |
| TME enriched | 42 | | 2.30 (1.97-5.42) | 0.06 | 0.43 (0.12-1.48) | 0.18 | 0.57 (0.07-4.60) | 0.60 | NA | NA |
| KMT2A | 39 | | 1.66 (0.6-4.8) | 0.35 | 0.9 (0.3-2.6) | 0.89 | 0.75 (0.09-6.04) | 0.78 | 1.79 (0.20-16.10) | 0.60 |
| MLLT10 | 31 | | 3.06 (1.07-8.73) | 0.04 | 1.15 (0.14-3.68) | 0.82 | 1.15 (0.14-9.52) | 0.90 | NA | NA |
| HOXA9-TCR | 21 | | 1.11 (0.23-5.21) | 0.90 | 0.93 (0.25-3.44) | 0.92 | 1.24 (0.15-10.4) | 0.84 | NA | NA |
| BCL11B | 18 | | 3.79 (1.04-13.8) | 0.04 | 0.53 (0.06-4.44) | 0.56 | NA | NA | NA | NA |
| **Pathway** | | | | | | | | | | |
| | | **European** | **African** | | **Admixed American** | | **East Asian** | | **South Asian** | |
| **Pathway** | Total number of patients | | OR (95% CI) | P value | OR (95% CI) | P value | OR (95% CI) | P value | OR (95% CI) | P value |
| Hematopoietic transcriptional | 1234 | Reference | 0.52 (0.27-1.03) | 0.05 | 0.72 (0.38-1.43) | 0.32 | 1.03 (0.30-6.50) | 0.97 | Infinity[b] | NA |
| Cell cycle | 991 | | 0.58 (0.40-0.87) | 0.007 | 0.73 (0.51-1.06) | 0.09 | 0.54 (0.28-1.07) | 0.07 | 0.80 (0.31-2.50) | 0.68 |
| Notch pathway | 956 | | 0.59 (0.41- | 0.007 | 0.92 (0.64- | 0.64 | 0.71 (0.37- | 0.32 | 0.74 (0.29- | 0.55 |

| | | | | | | | | | | |
|---|---|---|---|---|---|---|---|---|---|---|
| | | | 0.87) | | 1.33) | | 1.44) | | 2.13) | |
| Epigenetic | 791 | | 1.19 (0.82-1.74) | 0.36 | 0.95 (0.69-1.32) | 0.77 | 0.85 (0.46-1.62) | 0.62 | 0.78 (0.32-1.96) | 0.59 |
| Transcriptional regulation | 610 | | 1.15 (0.80-1.64) | 0.45 | 0.91 (0.66-1.25) | 0.55 | 0.87 (0.46-1.63) | 0.67 | 2.16 (0.88-5.81) | 0.10 |
| Jak | 379 | | 1.08 (0.72-1.58) | 0.71 | 0.85 (0.59-1.21) | 0.36 | 1.39 (0.71-2.63) | 0.32 | 1.07 (0.38-2.71) | 0.89 |
| PI3K | 373 | | 0.95 (0.63-1.41) | 0.81 | 0.83 (0.57-1.18) | 0.31 | 0.57 (0.30-1.36) | 0.30 | 1.32 (0.49-3.27) | 0.56 |
| Other | 332 | | 0.75 (0.48-1.13) | 0.19 | 0.70 (0.47-1.01) | 0.06 | 0.61 (0.26-1.28) | 0.22 | 0.65 (0.18-1.80) | 0.45 |
| Ras | 208 | | 1.68 (1.07-2.58) | 0.02 | 0.91 (0.57-1.41) | 0.67 | 1.32 (0.56-2.79) | 0.50 | 0.99 (0.22-3.00) | 0.99 |
| Signaling other | 197 | | 1.12 (0.68-1.77) | 0.66 | 0.74 (0.46-1.17) | 0.22 | 0.56 (0.16-1.41) | 0.27 | 0.59 (0.09-2.07) | 0.48 |
| RNA machinery | 175 | | 1.12 (0.66-1.84) | 0.65 | 1.01 (0.62-1.58) | 0.96 | 0.88 (0.30-2.11) | 0.80 | 2.18 (0.70-5.76) | 0.14 |
| Ribosome | 158 | | 0.28 (0.11-0.61) | 0.003[c] | 0.87 (0.52-1.38) | 0.57 | 0.50 (0.12-1.40) | 0.25 | 2.77 (0.96-7.07) | 0.04 |
| Cohesin | 102 | | 0.57 (0.23-1.20) | 0.18 | 1.14 (0.64-1.94) | 0.64 | 0.27 (0.02-1.28) | 0.20 | 0.59 (0.03-2.90) | 0.61 |
| Noncoding | 96 | | 1.10 (0.55-2.03) | 0.78 | 0.93 (0.49-1.66) | 0.82 | 0.60 (0.10-2.02) | 0.49 | 1.33 (0.21-4.77) | 0.71 |
| Protein modification | 93 | | 0.83 (0.36-1.70) | 0.64 | 1.09 (0.57-1.97) | 0.78 | 0.34 (0.02-1.63) | 0.30 | NA | NA |

[a] One multinomial model for subtype; separate logistic regression model for each pathway. Five subtypes and two pathways are not presented because of unstable estimates due to small numbers. NA indicates that model did not converge due to small numbers.

[b]All South Asian patients have this pathway alteration.

[c]Remained significant after adjustment for multiple comparisons for pathway analysis.

### Figures

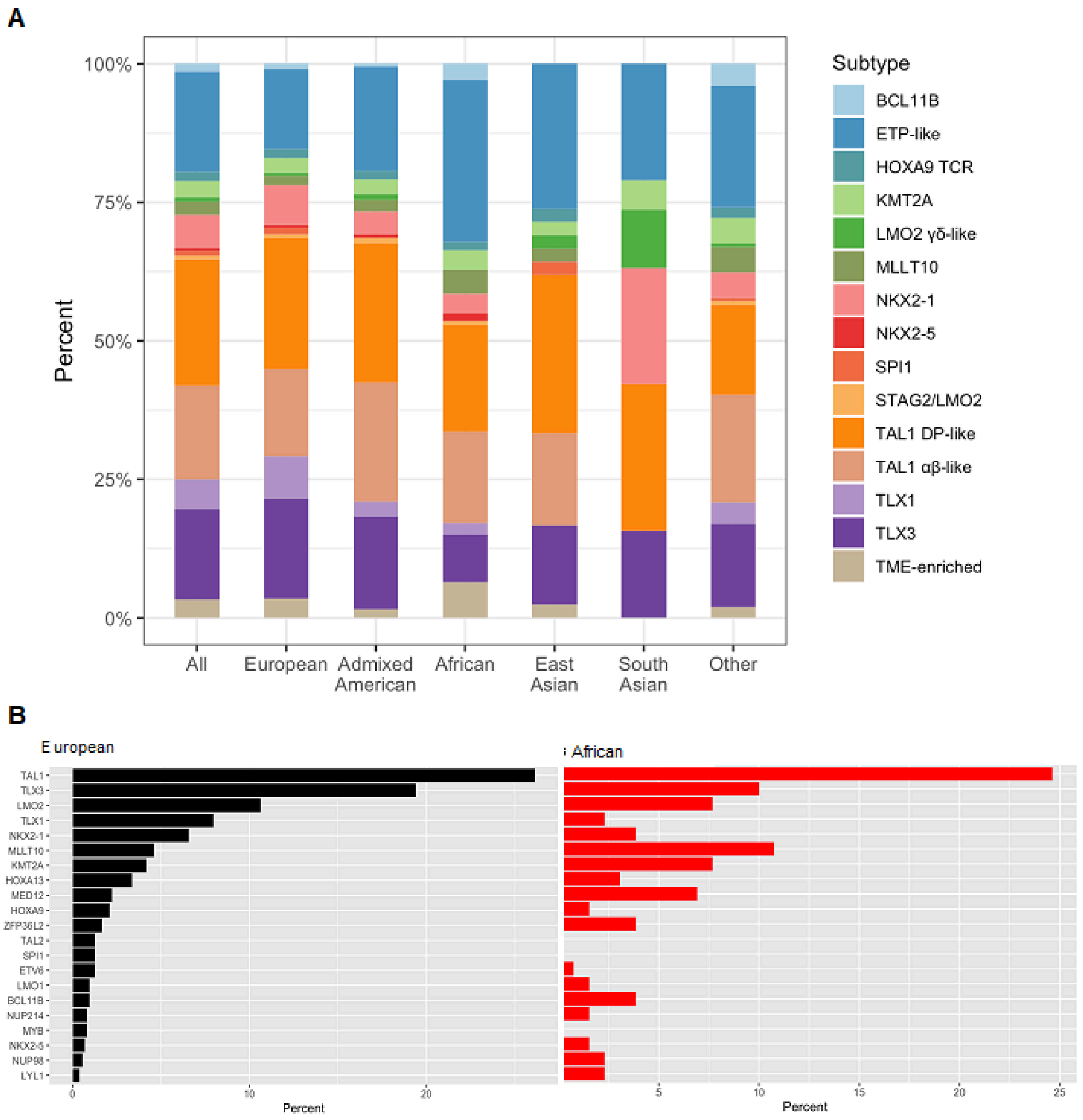

### Figure 1

T-cell Acute Lymphoblastic Leukemia (T-ALL) subtype and driver genes by genetic ancestry.

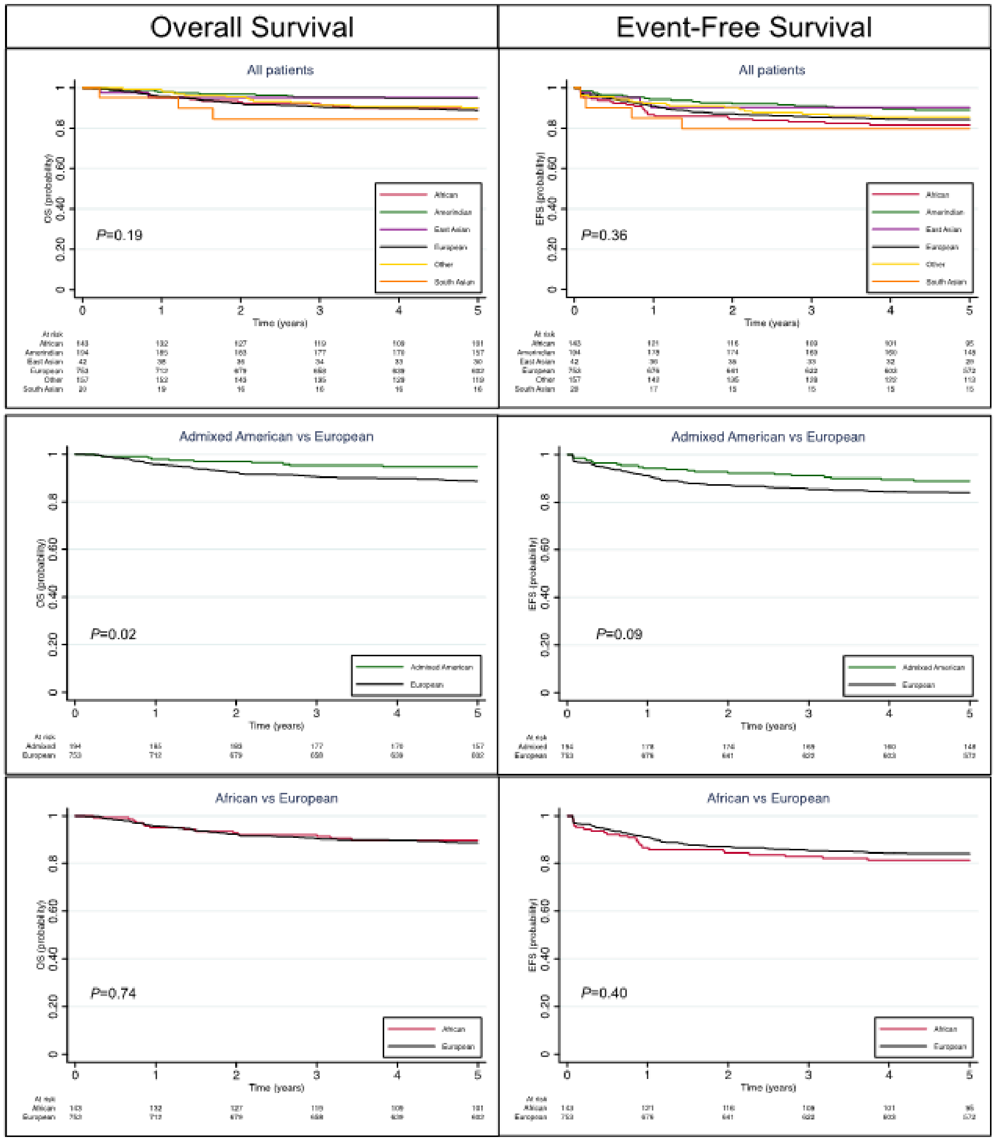

### Figure 2

Overall survival and event free survival by genetic ancestry.

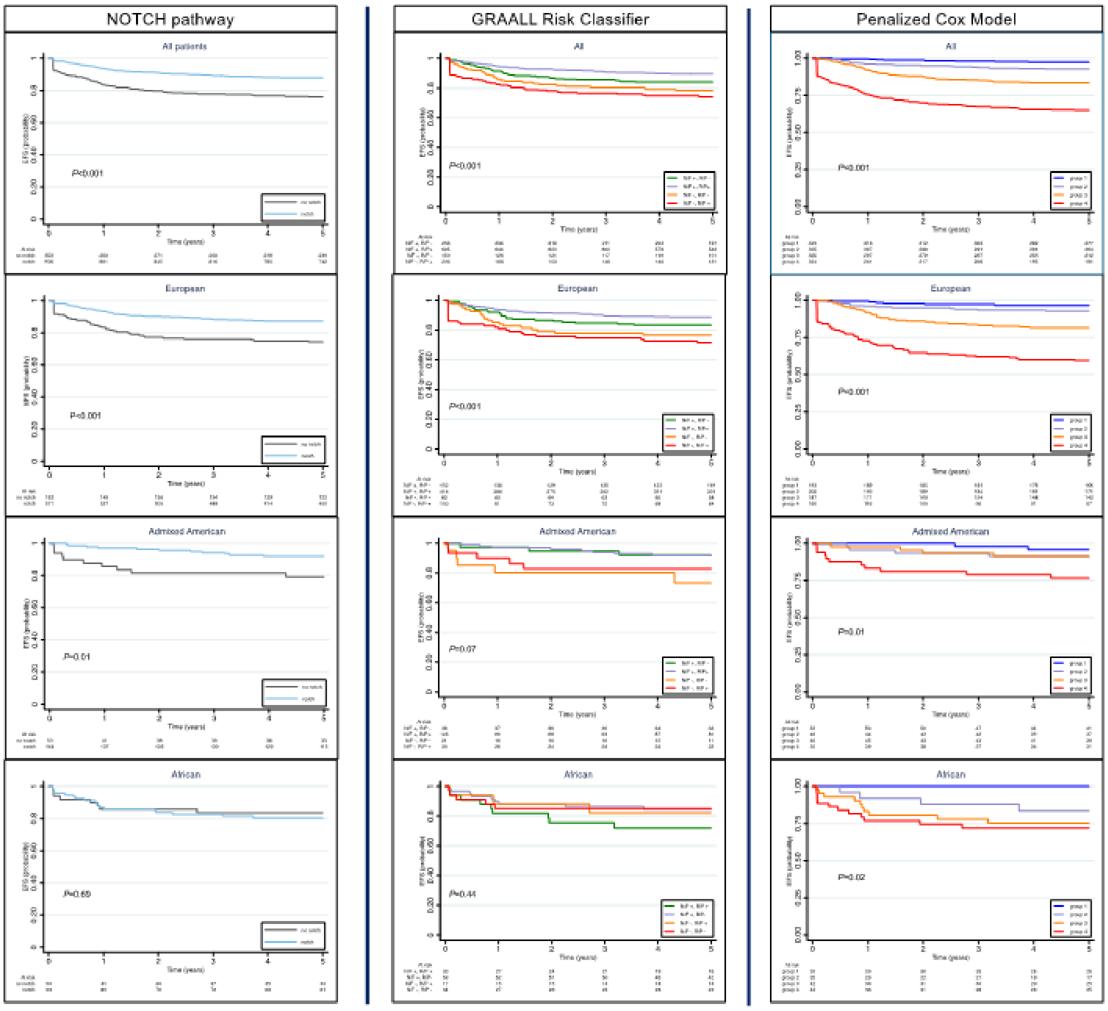

**Figure 3**

Event free survival by NOTCH pathway alteration and risk classifiers by genetic ancestry.

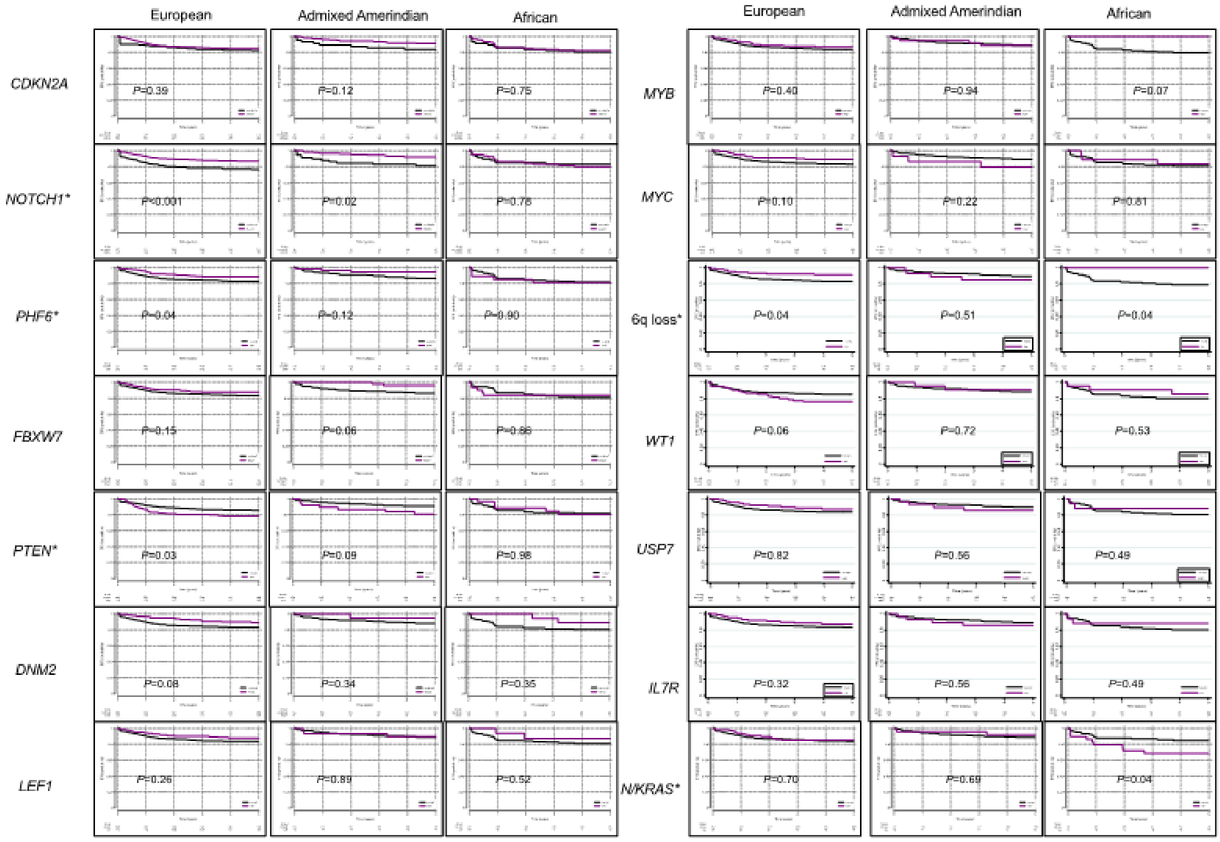

**Figure 4**

5-year event free survival for genes with at least 5% frequency for each ancestral group.

## Supplementary Files

This is a list of supplementary files associated with this preprint. Click to download.

- TALLANCESTRYSUPPLEMENT4AUG.docx
